# Epigenome-Wide Changes in the Cell Layers of the Vein Wall When Exposing the Venous Endothelium to Oscillatory Shear Stress

**DOI:** 10.3390/epigenomes7010008

**Published:** 2023-03-20

**Authors:** Mariya A. Smetanina, Valeria A. Korolenya, Alexander E. Kel, Ksenia S. Sevostyanova, Konstantin A. Gavrilov, Andrey I. Shevela, Maxim L. Filipenko

**Affiliations:** 1 Laboratory of Pharmacogenomics, Institute of Chemical Biology and Fundamental Medicine (ICBFM) SB RAS, Novosibirsk 630090, Russia; v.korolenia@g.nsu.ru (V.A.K.); alexander.kel@genexplain.com (A.E.K.); max@niboch.nsc.ru (M.L.F.); 2 Department of Fundamental Medicine, V. Zelman Institute for Medicine and Psychology, Novosibirsk State University (NSU), Novosibirsk 630090, Russia; 3 Department of Natural Sciences, Novosibirsk State University (NSU), Novosibirsk 630090, Russia; 4 Department of Research & Development, GeneXplain GmbH, D-38302 Wolfenbüttel, Germany; 5 Center of New Medical Technologies, Institute of Chemical Biology and Fundamental Medicine (ICBFM) SB RAS, Novosibirsk 630090, Russiashevela_ai@cnmt.ru (A.I.S.); 6 Laboratory of Invasive Medical Technologies, Institute of Chemical Biology and Fundamental Medicine (ICBFM) SB RAS, Novosibirsk 630090, Russia; 7 Department of Surgical Diseases, V. Zelman Institute for Medicine and Psychology, Novosibirsk State University (NSU), Novosibirsk 630090, Russia

**Keywords:** vein wall layers, endothelial cells, smooth muscle cells, fibroblasts, oscillatory shear stress, DNA methylation, gene regulation, master regulators

## Abstract

Epigenomic changes in the venous cells exerted by oscillatory shear stress towards the endothelium may result in consolidation of gene expression alterations upon vein wall remodeling during varicose transformation. We aimed to reveal such epigenome-wide methylation changes. Primary culture cells were obtained from non-varicose vein segments left after surgery of 3 patients by growing the cells in selective media after magnetic immunosorting. Endothelial cells were either exposed to oscillatory shear stress or left at the static condition. Then, other cell types were treated with preconditioned media from the adjacent layer’s cells. DNA isolated from the harvested cells was subjected to epigenome-wide study using Illumina microarrays followed by data analysis with GenomeStudio (Illumina), Excel (Microsoft), and Genome Enhancer (geneXplain) software packages. Differential (hypo-/hyper-) methylation was revealed for each cell layer’s DNA. The most targetable master regulators controlling the activity of certain transcription factors regulating the genes near the differentially methylated sites appeared to be the following: (1) HGS, PDGFB, and AR for endothelial cells; (2) HGS, CDH2, SPRY2, SMAD2, ZFYVE9, and P2RY1 for smooth muscle cells; and (3) WWOX, F8, IGF2R, NFKB1, RELA, SOCS1, and FXN for fibroblasts. Some of the identified master regulators may serve as promising druggable targets for treating varicose veins in the future.

## 1. Introduction

Varicose vein (VV) disease pathogenesis is of a multifactorial chronic nature; genetic, as well as epigenetic, factors make a considerable contribution to it and may serve as predisposing and affecting factors. Altered (non-uniform) shear stress, a tangential hemodynamic force, accompanies the pathological condition of the vessels and is one of the characteristics of many vascular disorders [1]. The mechanical effect of changes in hemodynamics may result in a cascade of molecular reactions leading to a shift in physiological processes and prompting the development of the disease. Not all factors underlying the initiation and the progression of VV disease have been discovered so far.

The three main layers constituting the vein wall are the following. The inner layer, the *tunica intima*, consists of a monolayer of endothelial cells with underlying collagen and elastin. The middle layer, the *tunica media*, predominantly consists of several layers of circularly located smooth muscle cells separated by collagen and elastin fibers. The outer layer, the *tunica adventitia* or *externa*, consists of collagen and, mainly, fibroblasts [2]. These layers are tightly connected to each other such that all changes happening to one layer are reflected in another layer. Moreover, these changes promptly spread through the whole vein wall, affecting its condition. Indeed, one of the shear-stress responsive genes, *LKLF* (*KLF2*), is endothelium-specific, and its expression affects tunica media formation and vessel wall integrity/stabilization [3].

Endothelial cells line the inner surface of the vein wall and, since they come in contact with both circulating blood components and surrounding tissues, they serve as primary sensors of the systemic, as well as the local, stimuli that may modulate the essential endothelial functions. For instance, due to blood flow, such modulation by local stimuli—hemodynamic forces—can lead to short-term vasoactive responses and long-term remodeling of the vessel wall [4]. Endothelial cells possess a set of mechanosensors, such as receptor tyrosine kinases, ion channels, integrins, and G-protein-coupled receptors that convert changes in the hemodynamics into the biochemical signals modulating endothelial cells’ gene expression, morphology, behavior, and phenotype through specialized mechanosensitive signaling pathways [1,5]. The endothelium plays a key role in vascular hemostasis, coagulation, inflammation, regulation of angiogenesis and vascular tone, and vascular permeability. Therefore, by sensing biochemical and biomechanical signals, as well as modifying its functional phenotype, the inner layer of the venous wall contributes to the maintenance of vascular homeostasis and the development of vascular pathology [4]. It is widely accepted that local hemodynamics play a crucial role in risk prediction [6].

Endothelial cells possess phenotypic and functional heterogeneity depending on the type of blood vessels, and, therefore, demonstrate vascular-bed-specific properties that define their response to fluid shear stress exposure (laminar, oscillatory, or pulsatile) [1]. There are different *in vitro* 2D and 3D (two-dimensional and three-dimensional) models mimicking the hemodynamics of the vascular system, but these cannot take into account all aspects of the complex *in vivo* environment, since various factors, including vessel geometry, blood viscosity and velocity, and blood pressures, affect hemodynamics in the vasculature. Nevertheless, these models are quite useful because they may help to investigate cellular responses and interactions under different patterns of fluid shear stress [6]. Of particular interest for us is the endothelial cells’ response to oscillatory shear stress (which is most effective for mimicking varicose vein conditions) and the transmission of the molecular signals to the adjacent layers of the vein wall. Thereby, the affected layers may respond to such molecular signals with changes within themselves and, subsequently, outside of themselves—into their environment. This is the case of the middle layer, which is responsible for constriction of the vessel wall and venous tone maintenance.

Studies have demonstrated that upon exposure to an altered hemodynamic environment, the vascular endothelium becomes activated (including ROS (reactive oxygen species) generation/production) and acquires a proinflammatory phenotype. This is caused by mechanoactivation of the NF-KB signaling pathway and characterized by the expression of endothelial inflammatory markers, augmented endothelial cell turnover, and increased endothelial cell apoptosis/loss [1,4,6].

Hemodynamic forces such as shear stress control the transcriptional activity of a large and diverse set of genes (noteworthily, not necessarily endothelium-specific genes) expressed by the vascular endothelium that plays a key role in transducing biomechanical forces into biochemical signaling [6,7]. We hypothesized that the oscillatory flow present in incompetent veins and primarily affecting endothelial cells leads to epigenomic changes in these cells and cells of other types constituting adjacent layers of the venous wall. The influence of epigenetic factors contributes to the multifactorial nature of varicose vein disease [8]. Such factors change the structure of DNA without changing the DNA sequence itself. Thus, DNA methylation can affect the transcription and, consequently, gene expression. Epigenomic modifications, i.e., epigenetic changes across the whole genome, are unique in terms of the type and amount of chemical modification at each location on a chromosome, and can vary from cell to cell; moreover, they are very dependent on environmental factors, so they can be modulated externally with small molecules [9]. This gives us hope that there could be awesome possibilities for the treatment of vascular diseases in the future.

“OMIC” approaches allow the profiling of multiple genes/their modifications and products simultaneously [10]. Recording “OMIC” data to measure gene activities, protein expression, and metabolic events is becoming a standard approach to characterize the pathological state of an affected tissue. Increasingly, several of these methods are applied in a combined approach, leading to large multi-“OMIC” data sets. Still, the challenge remains how to reveal the underlying molecular mechanisms that render a given pathological state different from the norm. The disease-causing mechanism can be described by a re-wiring of the cellular regulatory network, for instance as a result of epigenetic alterations influencing the activity of relevant genes. Reconstruction of the disease-specific regulatory networks can help to identify potential master regulators of the respective pathological processes [11,12,13,14]. Knowledge about these master regulators can point to ways to block a pathological regulatory cascade. Suppression of certain molecular targets as components of these cascades may stop the pathological process and cure the disease.

In case of the epigenome, and methylome in particular, epigenome-wide association studies (EWAS) significantly accelerated the field of epigenetics research. The aim of this study was to reveal epigenome-wide methylation changes in the cells-representatives of the venous wall layers, exerted by oscillatory shear stress towards the endothelium, which may result in the consolidation of gene expression alterations upon vein wall remodeling during varicose transformation. It is essential to investigate how hemodynamics is involved in VV disease initiation/progression, since it may render to the identification of potential drug targets in the molecular network that governs the studied pathological process. Yet, future mechanistic studies on the pathogenesis of the disease should provide new insights into potential targets for VVs treatment.

## 2. Results

### 2.1. Identification of Target Genes

In the first step of the analysis, target genes were identified from the uploaded experimental data. There were 300 genes (among all mapped to differentially methylated sites), with the highest number of transcription factor binding sites found in the regions ±400 bp from these methylated sites. Initially, raw methylation data were analyzed using Illumina GenomeStudio (Methylation Module) software, which mapped the differentially methylated sites to: (a) 36 differentially methylated genes (17 hypo- and 19 hypermethylated) for endothelial cells (ECs), (b) 92 differentially methylated genes (19 hypo- and 73 hypermethylated) for smooth muscle cells (SMCs), and (c) 353 differentially methylated genes (222 hypo- and 131 hypermethylated) for fibroblasts (FBs), as listed in Supplementary Tables S1–S3. This prompts us to speculate that the ratios of hypo- to hypermethylated genes may reflect possible changes after oscillatory shear stress exposure in overall transcription processes within the corresponding cell type of the vein layer: 0.89 < 1 (which means quenching) for ECs, 0.26 << 1 (which means considerable quenching) for SMCs, and 1.69 > 1 (which means activating) for FBs.

Then, we applied the software package “Genome Enhancer” (geneXplain platform) to a data set processed in GenomeStudio. The ultimate goal of this pipeline was to identify potential drug targets in the molecular network that governs the studied pathological process. According to the Genome Enhancer, there were twenty main genes (presented in Table 1) that changed their methylation statuses in ECs after being exposed to oscillatory shear stress.

After SMCs cells representing an adjacent (to the endothelium) vein wall layer—tunica media—were treated with cell culture media taken from +/− exposed ECs, they also changed their DNA methylation statuses (as shown in Table 2 for top ten hypo- and top ten hypermethylated genes).

After FBs representing an adjacent (to the tunica media) vein wall layer—tunica adventitia—were treated with cell culture media taken from +/− treated SMCs, they also changed their DNA methylation statuses (as shown in Table 3 for top ten hypo- and top ten hypermethylated genes).

**Figure 1:**
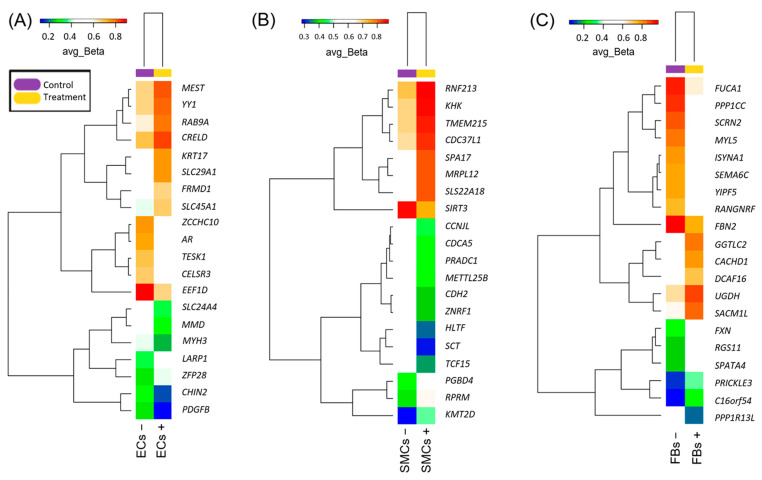
Cluster heatmap depicting methylation levels of the top 20 genes among those significantly hypo- and hypermethylated in 3 cell types upon exposure. (**A**–**C**) represent a heatmap for −/+ treated ECs, SMCs, and FBs, respectively. Heatmap displays differentially methylated genes ranging from hypomethylated (blue) to hypermethylated (red). avg_Beta represents an average methylation beta value and corresponds to a certain color within the range. Cluster pattern is shown on the left side of each diagram.

Epigenome-wide DNA methylation profiling revealed the changes in the cell type of each venous wall layer, not only in the venous endothelium exposed to oscillatory shear stress, but also in the cells-representatives of the adjacent layers. For a graphical illustration of the data shown in Table 1, Table 2 and Table 3, we created a cluster heatmap where the average beta values reflecting the methylation levels of the genes (assigned to CpG loci in the group of samples) are represented by colors, and the rows and columns of the data matrix have been ordered according to the output from clustering (Figure 1).

In Figure 1, a heatmap of differentially methylated genes is shown for each cell type separately. One can observe that there are clusters of genes that synchronously increase or synchronously decrease in methylation. Additionally, we combined all three sets of genes, each of them being differentially methylated in a certain cell type, with the corresponding beta values in all those cells (treated and untreated), and applied a cluster heatmap analysis in order to compare samples from different cells with each other (Supplementary Figure S1). Interestingly, the result was that they did not differ much (the difference between “treatment” and “control” in one cell type was often higher than the difference between control samples in other cell types). Additionally, in general, these genes had fairly stable methylation statuses in different cell types—they are represented with similar colors along the entire length of the heatmap.

However, we focused not just on the hypo-/hypermethylated sites, but rather on their regulators and potential master regulators. Differentially methylated sites were mapped to genes, and the top 300 genes with the highest number of transcription factor binding sites (found in the 400 bp regions around these methylated sites) were selected for further analysis.

### 2.2. Functional Classification of Genes

**Figure 2:**
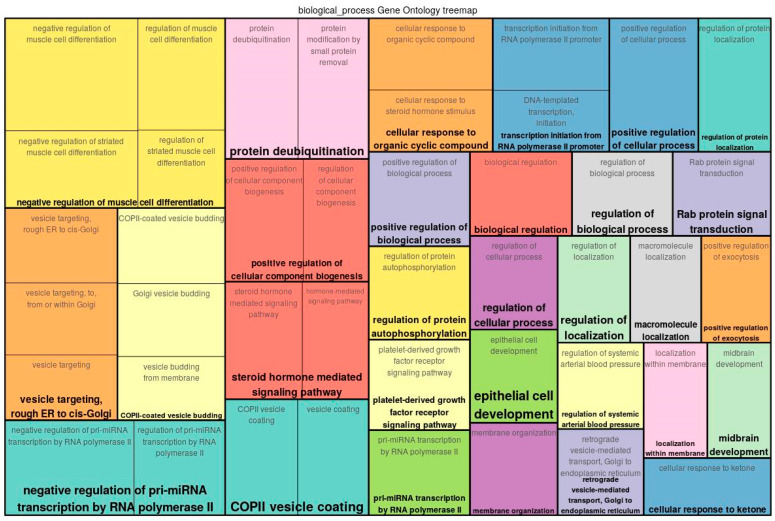
Enriched GO (biological process) tree map of the list of genes provided as input for ECs.

**Figure 3:**
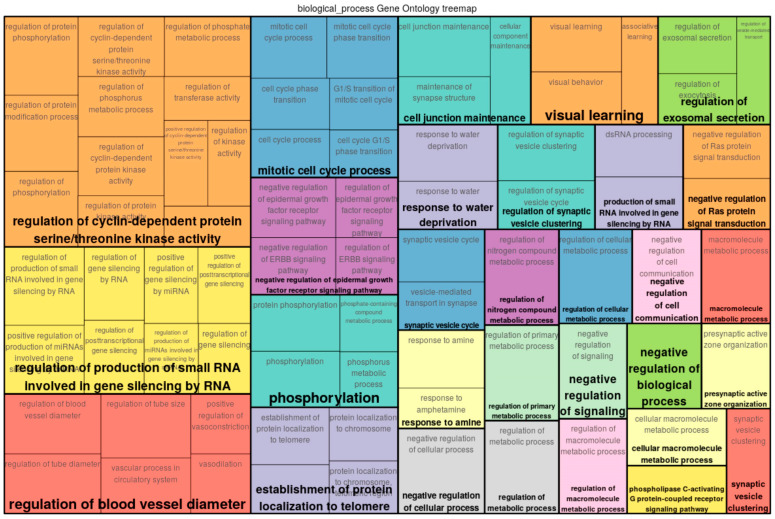
Enriched GO (biological process) tree map of the list of genes provided as input for SMCs.

**Figure 4:**
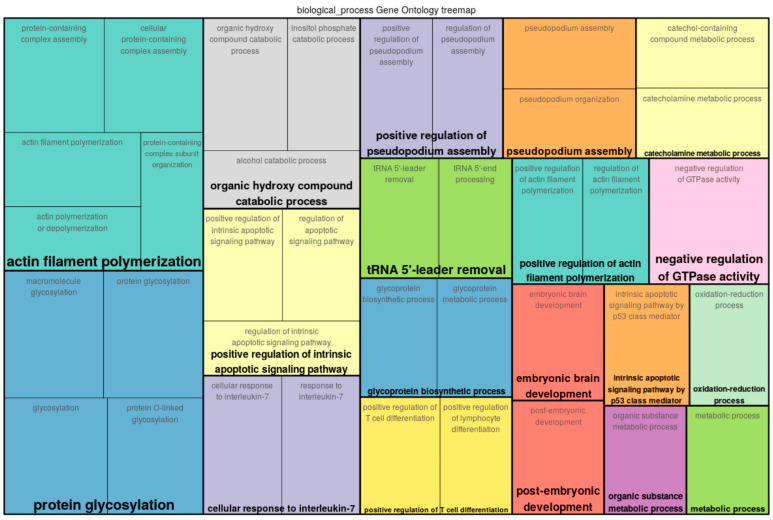
Enriched GO (biological process) tree map of the list of genes provided as input for FBs.

A functional analysis of the top 300 genes near differentially methylated sites was conducted by mapping the input genes to several known ontologies, such as the Gene Ontology (GO)_biological process and the ontology of signal transduction and metabolic pathways from the TRANSPATH^®^ database. Statistical significance was computed using a binomial test. Figure 2, Figure 3 and Figure 4 show the most significant categories for ECs, SMCs, and FBs, correspondingly.

GO analysis of the genes associated with differentially methylated sites showed that a number of genes potentially affected by differential methylation upon exposure of ECs to Oscillatory Shear Stress played roles in such processes as negative regulation of muscle cell differentiation (hit names: PDGFB, PLPP7, YY1), positive regulation of cellular component biogenesis (CCP110, HGS, TESK1, TIGD5, TPPP2), regulation of protein autophosphorylation (PDGFB, TESK1), platelet-derived growth factor receptor signaling pathway (HGS, PDGFB), steroid hormone mediated signaling pathway (AR, NR3C2, NR4A2), cellular response to organic cyclic compound (AR, LARP1, NR3C2, NR4A2, PDGFB), vesicle targeting/coating and rough ER to cis-Golgi and vesicle budding from membrane (CNIH2, RAB1B), positive regulation of exocytosis (HGS, RAB9A), regulation of systemic arterial blood pressure (AR, PDGFB), retrograde vesicle-mediated transport, Golgi to endoplasmic reticulum (RAB1B, RER1), macroautophagy (HGS, LARP1, RAB1B), etc. (see Supplementary Table S4).

Full classification of GO categories for SMCs may be seen in Supplementary Table S5. GO analysis revealed that a number of genes potentially affected by differential methylation upon exposure of SMCs to the preconditioned media from ECs (+/− oscillatory shear stress-exposed) played roles in such processes as negative regulation of cellular processes, regulation of nitrogen compound metabolic processes (hit names: C5AR2, CCND2, CD38, CD9, CDH2, CDKN3, CHFR, CHMP6, CLSPN, DCLK1, DDX5, etc.), regulation of metabolic process (C5AR2, CCND2, CCNJL, CCNT2, CCT7, HGS, etc.), negative regulation of cell communication (C5AR2, CD38, CDH2, DRD1, F2R, HGS, HIF1AN, ING2, P2RY1, PSMD13, SIRT3, SPRY2, etc.), regulation of the production of small RNAs involved in gene silencing by RNA (DDX5, LIN28A, TERT), presynaptic active zone organization (ERC2, PCDH17), regulation of protein phosphorylation (C5AR2, CCND2, CCNJL, CCNT2, CDH2, CDKN3, CHMP6, CLSPN, DRD1, F2R, HGS, MICAL1, NCKAP1L, P2RY1, etc.), regulation of blood vessel diameter and size (CD38, CPS1, DRD1, F2R, P2RY1), cell junction maintenance (ERC2, F2R, SYNGAP1), regulation of synaptic vesicle clustering (CDH2, PCDH17), regulation of cyclin-dependent protein serine/threonine kinase activity (CCND2, CCNJL, CCNT2, CDKN3), negative regulation of the epidermal growth factor receptor signaling pathway (CHMP6, HGS, SPRY2), vascular processes in the circulatory system (CD38, CPS1, DRD1, F2R, P2RY1), regulation of presynaptic cytosolic calcium ion concentration (ATP2B2, P2RY1), regulation of exosomal secretion (CHMP6, HGS), and smooth muscle contraction (CD38, DRD1, F2R).

HIF1AN (also known as FIH1)—a HIF1-alpha inhibitor—is the cellular oxygen sensor factor inhibiting hypoxia-inducible factor 1 alpha [15] by preventing its transcriptional activity and leading to adaptive responses to hypoxia. HIF1AN plays a critical role in controlling the survival of vascular ECs through interacting with Notch2 and repressing its activity [16]. This may point to an interconnection between ECs and SMCs belonging to adjacent layers. It is worth noting that the HIF1AN antagonist—HIF1-alpha—is not only regulated by the hypoxic stimulus, but can also act as a target for potentiating the protective effects from some adaptogenic triggers [17].

A full classification of GO categories for FBs may be seen in Supplementary Table S6. GO analysis revealed that a number of genes potentially affected by differential methylation upon exposure of FBs to the preconditioned media from SMCs (treated with the preconditioned media from +/− oscillatory shear stress-exposed ECs) played roles in such processes as negative regulation of GTPase activity (hit names: GPS1, IQGAP2, PTPRN2, RCC2), protein glycosylation (B3GALT6, DOLK, GALNT1, GALNT17, MAN2A2, RFNG, TET2, TMTC2, etc.), protein-containing complex assembly (CDC42EP2, CDC42EP4, CHMP6, F8, FMC1, GPX4, etc.), intrinsic apoptotic signaling pathway by p53 class mediator (PTTG1IP, RPL11, SHISA5, WWOX), actin filament polymerization (CDC42EP2, CDC42EP4, IQGAP2, MSRB2, PPP1R9A, TIGD5), the inositol phosphate catabolic process (IMPA2, NUDT3), the oxidation–reduction process (ADHFE1, CYP4B1, DHCR7, DHFR, F8, FAHD1, FXN, GPX4, HIF1AN, MSRB2, NDUFA7, NDUFS3.., etc.), and regulation of the apoptotic signaling pathway (FXN, INHBB, MCL1, NACC2, NDUFS3, NR4A2, PTTG1IP, RPL11, WWOX).

For instance, it was shown that activation of CDC42 is involved in the hypoxia-induced production of angiogenesis-promoting factors such as vascular endothelial growth factor (VEGF) [18], as well as in actin filament polymerization [19]. Proteins of this family also play roles in cytoskeletal remodeling and signaling, cell shape, directed migration and differentiation, and pathological fibroblast activation [20]. The aforementioned HIF1AN, participating in the oxidation–reduction process, is also present in FBs.

The results of the additional functional analysis of the input genes mapped to the ontology of signal transduction and metabolic pathways, according to the TRANSPATH^®^ database, are shown in Supplementary Figures S2–S4. The affected genes were significantly enriched with specific pathway ontology categories. A full classification of those categories for each cell type may be seen in Supplementary Tables S7–S9.

**Figure 5:**
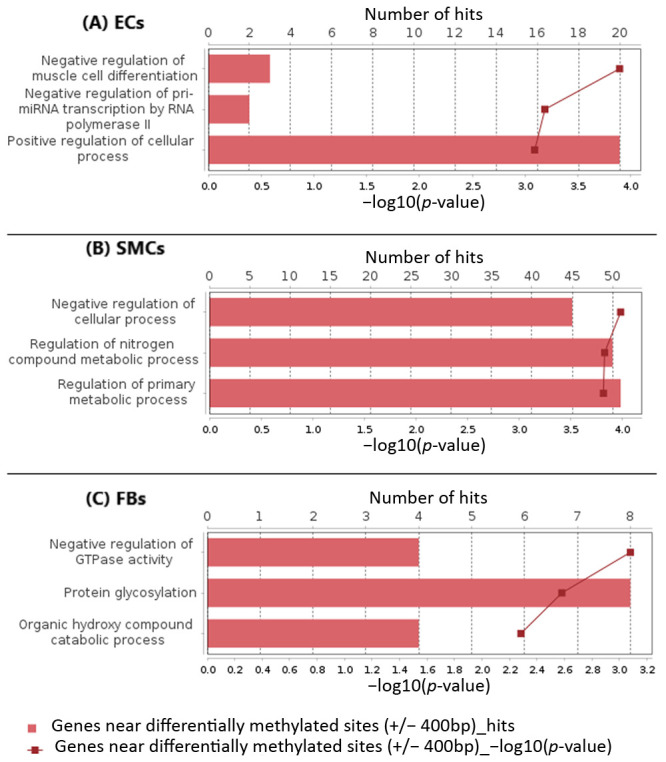
The most significant functional GO categories overrepresented among the observed genes near differentially methylated sites.

The result of overall GO analysis of the genes near differentially methylated sites can be summarized by the following diagram (Figure 5), which reveals the most significant functional categories overrepresented among the observed genes. Thus, we can report that our epigenome-wide analysis revealed important changes that accompany the effect of oscillatory shear stress on the venous endothelium, which spreads to SMCs and FBs that represent the middle and outer layers of the vein wall, correspondingly.

**Figure 6:**
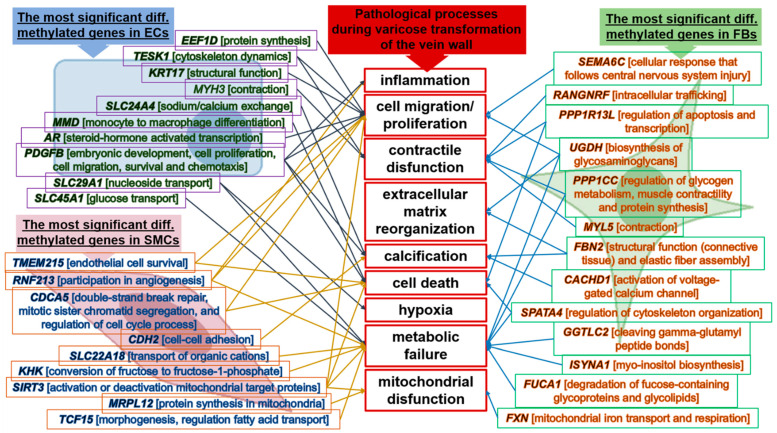
Association of genes (and their functions) differentially methylated in different cell types with the pathological processes during varicose transformation of the vein wall.

To better understand the relation of differentially methylated genes to the pathological condition, we developed an interactive illustration for the most significant of those genes in different cells and their cellular functions with respect to pathological consequences (Figure 6).

The figure shows how the functions of these genes (with the highest value of |DiffScore|) could be linked to the pathological processes involved in varicose transformation of the vein wall.

### 2.3. Analysis of Enriched Transcription Factor Binding Sites and Composite Modules

In the next step, a search for transcription factor binding sites (TFBS) was performed in the regulatory regions of the target genes by using the TF binding motif library of the TRANSFAC^®^ database. We searched for so-called composite modules acting as potential condition-specific enhancers of the target genes in their upstream regulatory regions (−1000 bp upstream of transcription start site (TSS)) and identified transcription factors regulating the activity of the genes through such enhancers.

Classically, enhancers are defined as regions in the genome that increase the transcription of one or several genes when inserted in either orientation at various distances upstream or downstream of the gene [21]. Enhancers typically have a length of several hundreds of nucleotides and are bound by multiple transcription factors in a cooperative manner [22].

In the current work, we used epigenomics data from the tracks (Supplementary Tables S1–S3) to predict the positions of potential enhancers regulating the genes near differentially methylated sites revealed by comparative epigenomics analysis. We took genomic regions −550 bp upstream and 550 bp downstream from the middle point of each interval of the track and checked whether these regions were located inside the 5 kb flanking areas of the genes near differentially methylated sites (or inside the bodies of the genes). In such cases, these genomic regions are used for the search for potential condition-specific enhancers. In all other cases, when the genes near differentially methylated sites did not contain epigenomic peaks in their bodies or in the 5 kb flanking regions, we used the upstream regulatory regions of these genes (−1000 bp upstream and 100 bp downstream of TSS) for our search for condition-specific enhancers.

We applied the Composite Module Analyst (CMA) method [8] to detect such potential enhancers as targets of multiple TFs bound in a cooperative manner to the regulatory regions of the genes of interest. CMA applies a genetic algorithm to construct a generalized model of the enhancers by specifying combinations of TF motifs (from TRANSFAC^®^) whose sites are most frequently clustered together in the regulatory regions of the studied genes. CMA identifies the transcription factors which, through their cooperation, provide a synergistic effect and, thus, have a great influence on the gene regulation process.

**Figure 7:**
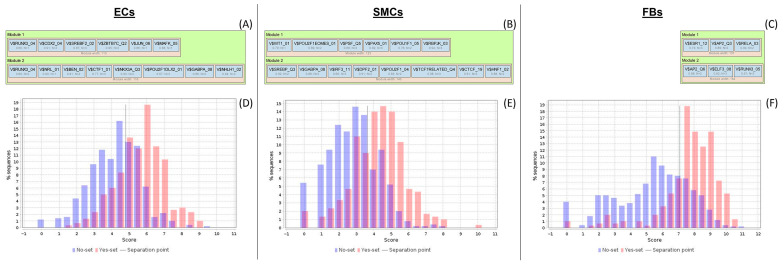
Enhancer model potentially involved in the regulation of target genes. (**A**–**C**)—the most specific composite modules obtained as the results of CMA analysis for ECs, SMCs, and FBs, correspondingly. “Module width” is the preferable distance between sites; “V$” stands for “vertebrates”; the right part of the PWM name represents the TF family name. Score of the best match is shown as the optimized cut-off of the PWM score; number of individual matches (N) for each PWM gives the maximal number of TF sites with the highest scores, which are computed in the module. (**D**–**F**)—for ECs, SMCs, and FBs, correspondingly, contain two histograms of the distributions of model scores (reflecting the number of TF site pairs found in the sequence) in the CpG regulatory regions (red) versus CpG sites of unchanged genes (blue). AUC = 0.76 for ECs and SMCs, and 0.81 for FBs.

To build the most specific composite modules, we chose genes as the input for the CMA algorithm. The results of this search are represented in Figure 7. The model consisted of two modules. In Figure 7A–C, the following information is shown for each module: PWMs (position weight matrixes) producing matches, scores of the best matches, and the number of individual matches (N) for each PWM. Through this analysis, we identified TFs whose binding to their control regions may be significantly altered by CpG methylation, leading to shifts in the expression of many genes in our experiment. The CMA algorithm identified enriched combinations of TFs with high statistical significance (Wilcoxon *p*-value = 2.33 × 10^−36^ for ECs, 4.34 × 10^−35^ for SMCs, and 1.62 × 10^−48^ for FBs). The AUC of the model for FBs achieved a value (=0.81) significantly higher than expected for a random set of regulatory regions (Z-score = 3.81), which means that there are significantly more TF site pairs in CpG regulatory regions compared to the background.

On the basis of the enhancer models, we identified transcription factors potentially regulating the target genes of our interest. We found 14, 17, and 7 transcription factors controlling the expression of target genes for ECs, SMCs, and FBs, correspondingly (see Table 4). In the table, ≤10 TFs for each cell type are shown. Full lists of TFs may be seen in Supplementary Tables S10–S12.

The key transcription factors which were predicted to be potentially regulating genes near differentially methylated sites in our experiment were: JUN, CDX2, and POU2F1 for ECs; SREBF2, LEF1, and IRF3 for SMCs; and RELA, ESR1, and TFAP2A for FBs. The relevance of these TFs is discussed later on, in the Section 3.

### 2.4. Finding Master Regulators in Networks

In the second step of the upstream analysis, common regulators of the revealed TFs were identified. We considered master regulators to be the keynodes with positive feedback loops; master regulator protein controls the activity of TFs which, in turn, activate the gene encoding the master regulator protein. The sorting of master regulators is conducted by total rank. The total rank is a kind of average rank that takes into account both whether this keynode is at the top of the regulatory pyramid (keynode score) and to what degree the gene encoding this keynode is regulated by predicted TFs (CMA score), as well as whether or not this gene contains a hypomethylation or hypermethylation site (epigenomics data). These master regulators appear to be the key candidates for therapeutic targets, as they have a master effect on the regulation of the intracellular pathways that activate the pathological processes which are the focal points of our study. The identified master regulators may be seen in Table 5, where the top 10 master molecules for ECs, SMCs, and FBs, correspondingly, are shown. Full lists of the master molecules for each type of cells may be seen in Supplementary Tables S13–S15.

**Figure 8:**
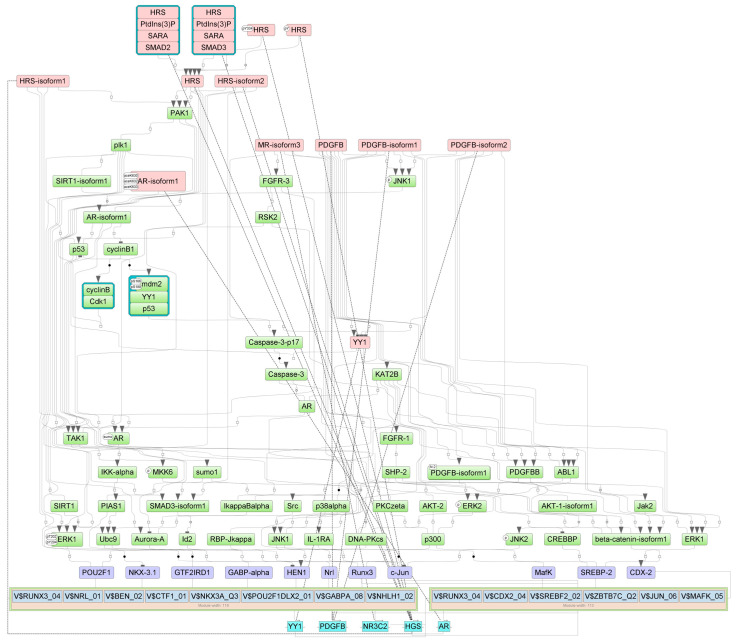
Diagram of intracellular regulatory signal transduction pathways of genes near differentially methylated sites in ECs. Master regulators are indicated by red rectangles, transcription factors are indicated by purple rectangles, and green rectangles represent intermediate molecules, which were added to the network during the search for master regulators from the selected TFs. Orange and blue frames highlight molecules that are encoded by genes mapped to differentially methylated sites. Positive feedbacks are represented by dotted lines.

**Figure 9:**
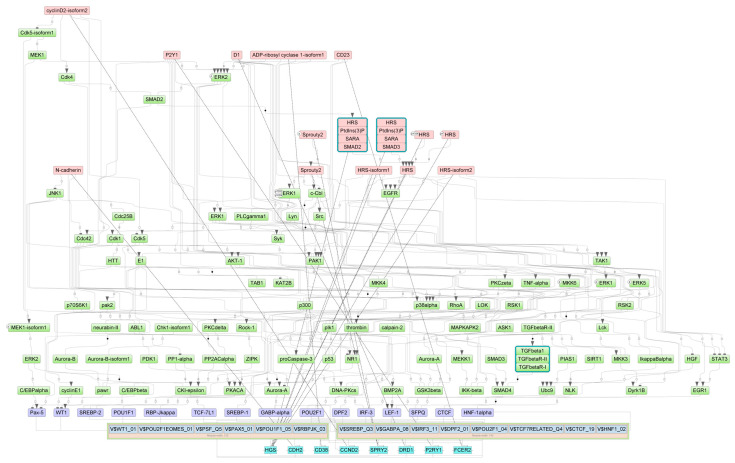
Diagram of intracellular regulatory signal transduction pathways of genes near differentially methylated sites in SMCs. Master regulators are indicated by red rectangles; transcription factors are indicated by purple rectangles; and green rectangles represent intermediate molecules, which were added to the network during the search for master regulators from the selected TFs. Orange and blue frames highlight molecules that are encoded by genes mapped to differentially methylated sites. Positive feedbacks are represented by dotted lines.

**Figure 10:**
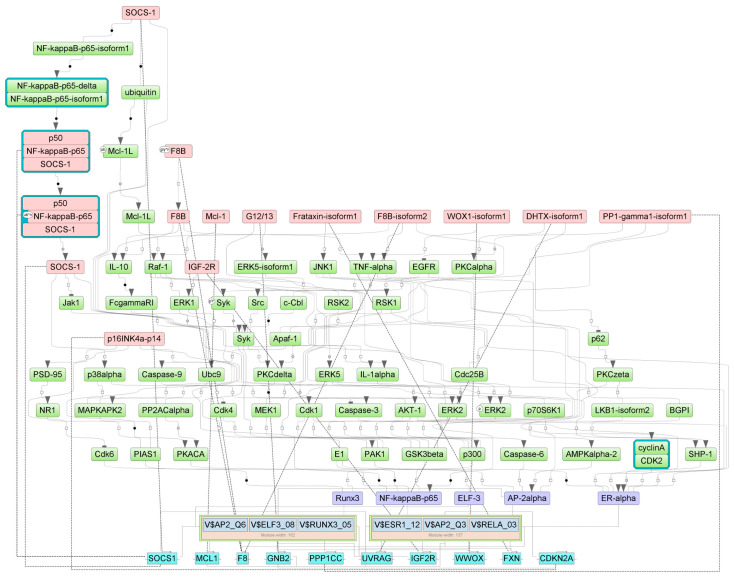
Diagram of intracellular regulatory signal transduction pathways of genes near differentially methylated sites in FBs. Master regulators are indicated by red rectangles, transcription factors are indicated by purple rectangles, and green rectangles represent intermediate molecules, which were added to the network during the search for master regulators from the selected TFs. Orange and blue frames highlight molecules that are encoded by genes mapped to differentially methylated sites. Positive feedbacks are represented by shown by dotted lines.

The intracellular regulatory pathways controlled by the aforementioned master regulators are depicted in Figure 8, Figure 9 and Figure 10, where positive feedback is represented by dotted lines. This diagram displays the connections between the identified TFs that play important roles in the regulation of genes near the differentially methylated sites and the selected master regulators that are responsible for the regulation of these TFs.

For ECs, master regulators HGS and PDGFB are involved in the platelet-derived growth factor receptor signaling pathway; AR and PDGFB are involved in epithelial cell development and regulation of systemic arterial blood pressure; and AR, HGS, and PDGFB are involved in the regulation of protein phosphorylation and cellular protein metabolic process (according to GO processes presented in Supplementary Table S4). Furthermore, the key pathways for master regulators in ECs are: the AR pathway; the IGF-1 pathway; MKK4 ---JNK1---/AR; PDGF A, PDGF B ---> AKT; PDGF B ---> STATs; PDGF B---/Ras; and the PDGF pathway (according to the pathway categories presented in Supplementary Table S7).

For SMCs, master regulators CDH2, HGS, and SPRY2 are involved in such key processes as regulation of the nitrogen compound metabolic process, regulation of cell population proliferation, and blood vessel morphogenesis. CDH2, HGS, P2RY1, and SPRY2 are involved in regulation of cellular metabolic process, negative regulation of cell communication, and phosphorylation. HGS and SPRY2 are involved in negative regulation of the ERBB signaling pathway, regulation of protein kinase activity, and regulation of the epidermal growth factor receptor signaling pathway. CDH2 and P2RY1 are involved in regulation of the synaptic vesicle cycle and neurogenesis. HGS, P2RY1, and SPRY2 are involved in positive regulation of gene expression; HGS and P2RY1 are involved in export from the cell and secretion by the cell (according to GO processes presented in Supplementary Table S5). The key pathways for the master regulators in SMCs are: Spry2 ---> ErbB1; EGF pathway; activin A ---> Smad3; activin A ---> Smad2; TGFbeta pathway; and N-cadherin ---Eplin---> actin; N-cadherin network (according to the pathway categories presented in Supplementary Table S8).

For FBs, the master regulators WWOX, F8, and FXN are involved in oxidation–reduction processes; FXN and WWOX are involved in regulation of the apoptotic signaling pathway (according to GO processes presented in Supplementary Table S6). The key pathways for master regulators in FBs are: Src ---/p73beta; ErbB4 mediated signaling; p73 pathway; IL-5 pathway; and SOCS-1 ---/STAT5; IL-2—STAT5 pathway (according to the pathway categories presented in Supplementary Table S9).

**Figure 11:**
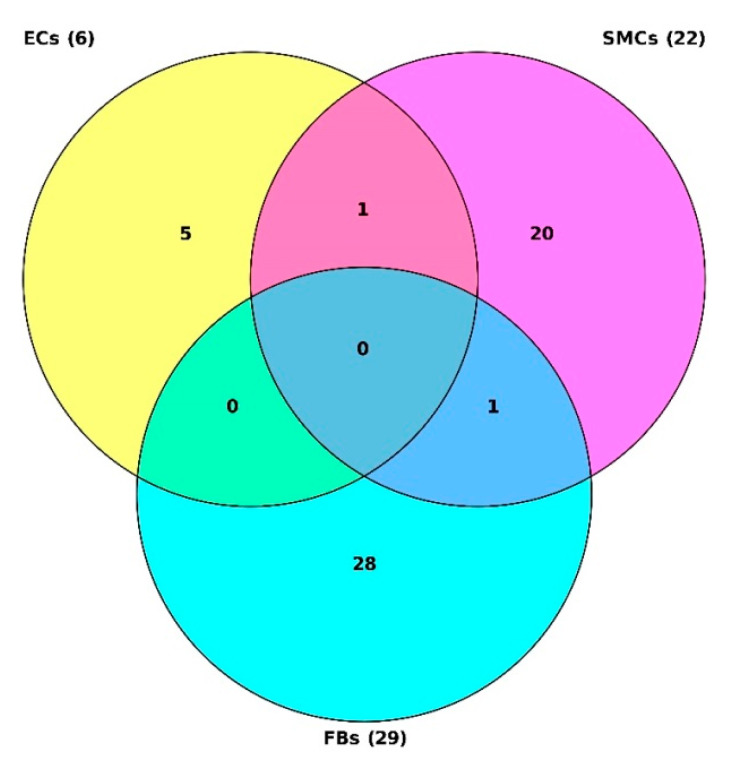
Venn diagram showing the intersection of 3 tables with top master regulators for ECs (yellow), SMCs (pink), and FBs (blue).

The Tables with master regulator molecules that have been converted into genes are Supplementary Tables S16–S18 for ECs, SMCs, and FBs, correspondingly. After we performed intersection of those three tables, we found that the first two cell types shared one potential master molecule {HRS(h),HRS(h){pY334},HRS(h){pY},HRS-isoform1(h),HRS-isoform2(h),HRS:PtdIns(3)P:SARA:SMAD2,HRS:PtdIns(3)P:SARA:SMAD3} that corresponded to potential master regulators HGS, SMAD2, SMAD3, and ZFYVE9 (see the Venn diagram in the Figure 11). The second two cell types—SMCs and FBs—representing the middle and outer layers of the venous wall, correspondingly, also shared one potential master molecule {ZNRF1(h),ZNRF1-isoform1(h),ZNRF1-isoform2(h} that corresponded to the potential master regulator ZNRF1. In addition, ZNRF1 was in the fourth place (according to the DiffScore = −30.56, *p*-value < 0.001) among the genes hypomethylated in SMCs upon our treatment, and this gene was also hypomethylated in FBs, albeit to a lesser extent (DiffScore = −22.02).

**Figure 12:**
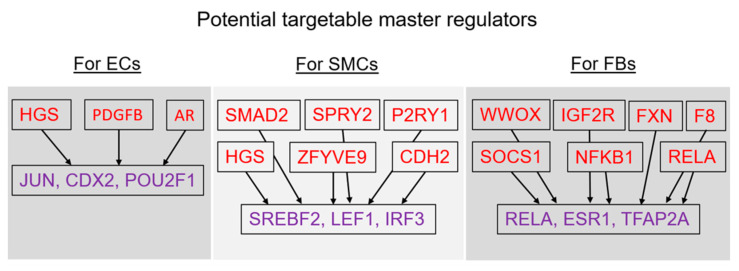
Potential targetable master regulators for each cell type representing the layers of the vein wall.

For the final summary, Genome Enhancer software chose the potential master regulators that were the most interesting and promising in terms of druggability. These master regulators control the activity of certain TFs regulating the genes near differentially methylated sites. Thus, the most targetable master regulators appeared to be the following: (1) HGS, PDGFB, and AR (corresponding to the master molecules {HRS}, {PDGFB}, and {AR-isoform1}), which control the activity of transcription factors JUN, CDX2, and POU2F1 for endothelial cells; (2) HGS, CDH2, SPRY2, SMAD2, ZFYVE9, and P2RY1 (corresponding to the master molecules {HRS}, {N-cadherin}, {Sprouty2}, {HRS:PtdIns(3)P:SARA:SMAD2}, and {P2Y1}), which control the activity of transcription factors SREBF2, LEF1, and IRF3 for smooth muscle cells; and (3) WWOX, F8, IGF2R, NFKB1, RELA, SOCS1, and FXN (corresponding to the master molecules {WOX1}, {F8B}, {IGF-2R}, {p50:NF-kappaB-p65:SOCS-1}, {FXN}, and {SOCS-1}), which control the activity of transcription factors RELA, ESR1, and TFAP2A for fibroblasts (see Figure 12).

## 3. Discussion

This study is the first attempt, to the best of our knowledge, to assess the changes to methylation profiles in the cells representing the corresponding layers of the vein wall in response to oscillatory shear stress, which somehow reflects the events happening in incompetent veins (VVs). Such an effort to resolve the heterogeneity of cell composition of the vein as an organ may deliver new insights into the mechanism of VV pathogenesis. Our 2D experimental model may not have been 100 percent realistic, but it definitely covered some aspects of the pathological condition. It is worth mentioning that our experimental design, which utilized oscillatory shear stress, partially represented proatherogenic conditions that are characterized by low-magnitude and oscillatory shear stress [1].

The cellular monolayer lining the *tunica intima* is normally subjected to biomechanical stimuli resulting from shear stress and from strain due to stretching of the vein wall. Shear stress has been implicated in altering the structure and functional properties of ECs at the cellular and molecular levels, with profound effects on physiology [23]. The vascular endothelium *in vivo* acts as a signal transduction interface for hemodynamic forces which determine the cytoskeletal organization, shape, and function of ECs, allowing the vessels to cope with physiological or pathological conditions [24]. This must be true for conditions *in vitro*. Interestingly, in response to shear stress, ECs increase NO production leading to an enhancement of the shear stress response of leukocytes [25]. On the other hand, we cannot exclude the outside-in hypothesis, according to which the *tunica adventitia* may be a sensor of vascular wall disruption and dysfunction, as well as an early responder and activator of the blood vessels’ response to injury [26]. The *tunica media* is in between, and must react and adjust to all possible effects from the inside and outside of the vein wall.

In this work, we have shown that the exposure of the venous endothelium to oscillatory shear stress not only resulted in epigenome-wide changes within this layer, but exerted even more prominent changes in the neighboring layers of the vein wall. Moreover, we observed different ratios of hypo- to hypermethylated genes, which may reflect possible changes caused by oscillatory shear stress in the overall transcription processes within the corresponding cell type of the vein layer: quenching for ECs (0.89 < 1), considerable quenching for SMCs (0.26 << 1), and activation for FBs (1.69 > 1). In turn, this may overlap with morphological changes during the varicose transformation of the venous wall, when endothelial dysfunction, impairment of the functional smooth muscle layer, and accrescence of adventitia are often observed. It is amazing how an impact of oscillatory shear stress on the inner layer alone is capable to launch much bigger changes in the adjacent layers of the vessel.

In the current work, we were limited by the methylation studies, but a combination of DNA methylation with gene expression could provide much more information about the molecular mechanisms involved. Herein, we used 27 K CpG arrays that represented only a fraction of the CpG positions in the genome (located near gene bodies and covering mainly predominately invariant methylation regions) that can become methylated and potentially affect gene regulation; thus, other regions not covered by these arrays were not included in our analysis. Despite this, we were able to discover a number of differentially methylated CpGs mapped to the genes whose functions were linked to the transformative changes that occurred in the vein wall (Figure 6). In addition, in our study, we conducted an analysis of the individual DMSs (differentially methylated sites) located in various genes, but we did not analyze DMRs (differentially methylated regions) that could potentially provide additional information about epigenetic changes in the genome and draw attention to some specific genes, though DMRs usually have nothing to do with gene regulation.

Conventional approaches of statistical “OMICs” data analysis provide only very limited information about the causes of the observed phenomena, and, therefore, contribute little to the understanding the pathological molecular mechanism. In contrast, the “upstream analysis” method [11,12,13,14] applied herein is designed to provide a casual interpretation of the data obtained for a pathology state. This approach comprises two major steps: (1) analyzing promoters and enhancers of differentially expressed genes for the transcription factors (TFs) involved in their regulation and, thus, important for the process under study; (2) reconstructing the signaling pathways that activate these TFs and identifying master regulators at the top of such pathways. For the first step, the database TRANSFAC^®^ [27] is employed together with the TF binding site identification algorithms Match [28] and CMA [21], so that pipeline discovers TFs that regulate genes’ activities in a pathological state. The activities of these TFs are controlled by so-called master regulators, which are identified in the second step of analysis. The second step involves the signal transduction database TRANSPATH^®^ [22] and special graph search algorithms [29] implemented in the “Genome Enhancer” software. After a subsequent druggability checkup, the most promising master regulators are chosen as potential drug targets for the analyzed pathology.

In the present work, we have revealed the master regulators that control the activity of certain TFs regulating genes near the differentially methylated sites for ECs, SMCs, and FBs, which represent the three layers of the venous wall. Herein, we will discuss only those potential master regulators which are the most promising in terms of druggability [30].

JUN, CDX2, and POU2F1 have been revealed as the key transcription factors predicted to potentially regulate genes near differentially methylated sites in our experiment for ECs exposed to oscillatory shear stress. JUN (Jun proto-oncogene, AP-1 transcription factor subunit) is a member of the Jun family of proteins, which are primary components of the activating protein transcription factor [31]. It is inducible by hypoxia related to endothelial cell barrier dysfunction [32]. CDX2 (caudal-type homeobox-2) is a member of the caudal-related homeobox transcription factor gene family. Aberrant expression of the *CDX2* gene is associated with intestinal inflammation [33]. Researchers hypothesize that it is related to the reconstruction of the blood vessels [34]. POU2F1 (POU class 2 homeobox 1) is shown as a transcription factor regulated by DNA damage [35] and as a transcriptional repressor for genes expressed in ECs [36]. The *POU2F1* gene may play an important role in the development of primary VVs [37], as well as generally, in the condition of the vascular system [38].

The most obvious master regulators of the aforementioned TFs were HGS, PDGFB, and AR. HGS (hepatocyte growth factor-regulated tyrosine kinase substrate) is involved in tight junction protein trafficking and ECs permeability [39]. HGS is necessary for maintaining cerebrovascular stability [40]. PDGFB (platelet-derived growth factor subunit B) is expressed at a very low level in healthy vessels [41]. It contributes to the migration and proliferation of SMCs [42] and plays a role in cell growth, apoptosis, and actin reorganization [41]. It is believed that platelet-derived growth factor is necessary for *tunica intima* growth and to prevent regression of its thickening [43]. Platelet-derived growth factors are involved in tissue homeostasis regulation due to control of the interstitial fluid pressure [44]. ARs (androgen receptors) play a role in vascular calcification [45], vascular SMC migration [46], endothelial dysfunction [47,48], and induction of vascular SMC apoptosis [49]. In veins from organ donor extraction (from patients without VVs) ARs were located in the adventitia. The redistribution of ARs through the venous wall was observed in VV conditions, and as a result, AR-positive cells were found in the neointima [50].

SREBF2, LEF1, and IRF3 have been revealed as the key transcription factors predicted to potentially regulate genes near differentially methylated sites in our experiment with SMCs treated with culture media from ECs ± exposed to oscillatory shear stress. In vascular endothelial cells, SREBF2 (sterol regulatory element binding transcription factor 2) is activated by sterol loss [51] and oscillatory shear stress [52]. It also promotes TGF-β1-induced cell movement [53]. LEF1 (lymphoid enhancer binding factor 1) plays an important role in embryogenesis and tumorigenicity [54]. LEF1 suppresses the expression of epithelial/endothelial–mesenchymal transition-relevant genes, which contributes to the malignancy of colonic adenocarcinomas [55]. IRF3 (interferon regulatory factor 3) is member of a family of transcription factors for genes associated with innate and adaptive immune responses [56]. In response to low shear stress, IRF3 is activated, which leads to endothelial inflammation [57].

For SMCs, the most obvious master regulators of the TF identified were HGS, CDH2, SPRY2, SMAD2, ZFYVE9, and P2RY1. CDH2 (cadherin 2) is essential for vascular SMC survival [58]. Inhibition of CDH2 function retards SMC migration and the promotion of ECs survival [59]. Blockade of SPRY2 (sprouty RTK signaling antagonist 2) (together with blockade of Dll4) leads to augmentation of the expression of venous markers in arteries [60]. *SPRY2* is upregulated in response to fibroblast growth factor 2 in primary dermal ECs [61]. SMAD2 (SMAD family member 2) mediates the signal of the transforming growth factor beta, which allows for the regulation of cell proliferation, apoptosis, and differentiation. Low fluid shear stress activates SMAD2, leading to inward remodeling in atherosclerotic vessels [62]. ZFYVE9 (zinc finger FYVE-type containing 9) participates in the transforming growth factor beta signaling pathway. ZFYVE9 recruits the aforementioned master regulator SMAD2 to the transforming growth factor beta receptor complex by controlling its subcellular localization [63]. P2RY1 (purinergic receptor P2Y1) functions as a receptor for extracellular ATP and ADP. The expression of the *P2Y1* in vascular ECs has also been shown [64]. It has previously been demonstrated that P2Y1 mediates ADP stimulation of MAPK pathways and ECs migration [65].

RELA, ESR1, and TFAP2A have been revealed as the key transcription factors predicted to potentially regulate genes near differentially methylated sites in our experiment with FBs exposed to culture media from pretreated SMCs. RELA (RELA proto-oncogene, NF-kB subunit) is a member of the NF-kB family [66]. The NF-kB pathway can be activated by different stimuli, including cytokines, oncogenes, oxidative stress, and DNA damage [67,68]. RELA inhibition led to the inactivation of proinflammatory molecules [69]. *ESR1* (estrogen receptor 1) gene expression in the VVs of women around menopause noticeably increases. ESR1 is present in the endothelium, SMCs, and some adventitial cells in the femoral veins [70]. TFAP2A (transcription factor AP-2 alpha) is expressed in the neural tube, neural crest, facial prominences, and limb bud mesenchyme throughout embryogenesis [71]. In ECs, TFAP2A plays a role in cell proliferation [72].

For FBs, the most obvious master regulators of the TF identified were WWOX, F8, IGF2R, NFKB1, RELA, SOCS1, and FXN. WWOX (WW domain containing oxidoreductase) is involved in cell proliferation, differentiation, and metabolism [73]. Mutations in the *WWOX* gene cause neurodevelopmental and brain degenerative disorders [74]. F8 (coagulation factor VIII) participates in the intrinsic pathway of blood coagulation. Defects in this gene result in hemophilia A, a common recessive X-linked coagulation disorder [75]. Activation of cardiac IGF2R (insulin like growth factor 2 receptor) results in cardiomyocyte hypertrophy, cardiomyocyte proliferation, binucleation, or apoptosis [76]. *NFKB1* (nuclear factor kappa B subunit 1) gene mutants affect the expression of mitochondrial morphology-related proteins, leading to excessive mitochondrial fission [77]. SOCS1 (suppressor of cytokine signaling 1) is a member of the STAT-induced STAT inhibitor. A decrease in SOCS1 promotes immune activation of SMCs [78]. FXN (frataxin) is a mitochondrial protein [79] expressed mainly in tissues with high metabolic rates (e.g., heart and brown fat) [80].

After three tables showing the most promising master regulators for each cell type (Tables S16–S18, where all master molecules were converted into genes) were intersected, it was revealed that ECs and SMCs share such potential master regulators as HGS, SMAD2, SMAD3, and ZFYVE9, and SMCs and FBs share a potential master regulator—ZNRF1. It is known that rs17684886 in *ZNRF1* is associated with diabetic retinopathy [81], and its expression is induced in peripheral nerves after injury [82], so its overexpression causes neurite-like elongation. It has been found that expression of the SMAD2 protein is progressively increased in reactive lesions and oral submucous fibrosis (OSMF) [83], and SMAD3 contributes to ascending aortic dilatation independently of transforming growth factor-beta in bicuspid and unicuspid aortic valve disease [84], which is consistent with our data.

A meta-analysis of epigenome-wide association studies in trauma-exposed cohorts revealed the association of the *HGS* differential methylation in whole blood-derived DNA with post-traumatic stress disorder [85]. Recently, a novel physiological role of endogenous HGS—a key component of the endosomal sorting complex required for transport (ESCRT)—has been explored in the vascular system. It was discovered that in mice, knockout of this gene in brain ECs led to impaired endothelial apicobasal polarity and brain vessel collapse; thus, the product of this gene was essential for vascular endothelial (VE)-cadherin recycling to the plasma membrane, pointing to a crucial function of HGS in the maintenance of endothelial cell polarity and cerebrovascular stability [40]. All of these studies appear to be supportive of the data analyzed in this study.

## 4. Materials and Methods

### 4.1. Sample Preparation and Cell Culture Experiments

Non-varicose great saphenous vein segments (adjacent to varicose vein segments) left after surgeries on 3 patients with VVs (C2-C3 CEAP [86] clinical classes) were immediately placed in cell culture media and transported to the laboratory for the subsequent production of primary cell cultures. Mechanically crushed fragments of the vein segments were treated with a solution of type II collagenase. A growth medium was added to the obtained suspensions of pieces and cells and centrifuged for 5 min at 300× *g*; then, the supernatant was taken and the sediment was resuspended in the medium for endothelial growth before being seeded on adhesive plates coated with type IV collagen. Pieces with cells (in the medium, but not covered with it, to prevent floating) were cultured under conditions of 5% CO_2_, 37 °C. When there was a sufficient density of cells that had grown from the pieces (in reality, this was a mixture of endothelial cells, smooth muscle cells, and fibroblasts), they were removed from the plastic with a TrypLE Express solution (Life Technologies, Carlsbad, CA, USA) and sorted by magnetic immunosorting using a CD31 MicroBead Kit (Miltenyi Biotec) according to the manufacturer’s instructions. CD31+ endothelial cells were seeded in endothelial growth medium (EGM-2 Endothelial Medium, Lonza, Basel, Switzerland), CD31− cells were seeded in the growth media for SMCs and FBs (SmGM-2 Smooth Muscle/FGM-2 Fibroblast BulletKits, Lonza), respectively, on adhesive plates coated with type IV collagen (primary cell cultures are illustrated in Supplementary Figure S5). The endothelial cells grew much more slowly compared to SMCs and FBs.

ECs were either exposed to oscillatory shear stress for 1 day (using a Multitron Cell shaker-incubator (INFORS HT) at 37 °C and 5% CO_2_, with platform oscillation only in the plane along the XY axis) or left in static conditions (37 °C and 5% CO_2_). Then, SMCs were treated for 1 day with preconditioned media from ECs, and FBs were subsequently treated for 1 day with preconditioned media from SMCs. Each experimental condition was performed in triplicate. After every exposure, the cells were harvested and subjected to DNA isolation using TRIzol Reagent (Life Technologies, Carlsbad, CA, USA). The experiment design is schematically shown in Supplementary Figure S6. DNA from all samples was further processed according to the manufacturer’s instructions (Illumina, Inc., San Diego, CA, USA), and then taken for methylation microarray analysis.

### 4.2. Epigenome-Wide DNA Methylation Analysis

DNA methylation microarray analysis was carried out according to the standard Illumina protocol. A total of 1 μg of gDNA was bisulfite converted using a EZ DNA Methylation™ Kit (Zymo Research, Irvine, CA, USA) according to the manufacturer’s protocol. After that, for genome-wide screening of methylation events, we used Infinium HumanMethylation27 BeadChips (Illumina), which cover 27,578 CpG sites spanning 14,495 genes per sample. Arrays were scanned on the Illumina iScan. Overall chip performance and the quality of the raw data were checked using Illumina GenomeStudio (methylation module) software in accordance with the manufacturer’s instructions (GenomeStudio Methylation Module v1.8 User Guide [87]). The raw intensity data were quantile-normalized. The methylation level of each CpG locus was calculated as methylation beta-value (β = intensity of the methylated allele (M)/(intensity of the unmethylated allele (U) + intensity of the methylated allele (M) + 100). Differential methylation (hypo- or hypermethylation) of the CpG sites was determined based on a DiffScore cut-off of ±13. DiffScore is the measure of the likelihood of variability between the compared groups. It is directly derived from the *p*-value, for which it provides methylation change directionality. It is a log10 transformation of the *p*-value and provides the *p*-value with scale and direction: *p*-value = [10^(DiffScore/10)] for hypomethylated genes, and [10^(−DiffScore/10)] for hypermethylated genes. The higher (or lower) the DiffScore is, the more likely it is that a change in methylation has taken place. Statistically significant values were determined (*p*-value < 0.05 that corresponds to |DiffScore| > 13): DiffScore < 0 corresponds to hypomethylated genes; DiffScore > 0 corresponds to hypermethylated genes. Differentially methylated (DM) sites were associated with genes using the Custom Model of the Illumina GenomeStudio. Then, the software package “Genome Enhancer” from the geneXplain platform was applied to a data set analyzed with GenomeStudio and Microsoft Excel. A cluster heatmap analysis was performed within the geneXplain platform.

### 4.3. Advanced Bioinformatics Analyses

Transcription factor binding sites (TFBS) in promoters and enhancers of genes near differentially methylated sites were analyzed using known DNA-binding motifs described in the TRANSFAC^®^ library, release 2022.2 (geneXplain GmbH, Wolfenbüttel, Germany) (https://genexplain.com/transfac (accessed on 22 December 2022)). The motifs were specified using position weight matrices (PWMs), which gave weights to each nucleotide in each position of the DNA-binding motif for a TF or a group of them.

We searched for TFBS that were enriched in the promoters and enhancers under study as compared to a background sequence set, such as promoters of genes that were not differentially regulated under the conditions of the experiment. We denoted study and background sets briefly as Yes and No sets. In the current work, we used a workflow considering promoter sequences of a standard length of 1100 bp (−1000 to +100). The error rate in this section of the pipeline was controlled by estimating the adjusted *p*-value (using the Benjamini–Hochberg procedure) in comparison to the TFBS frequency found in randomly selected regions of the human genome (adjusted *p*-value < 0.01).

We applied the CMA (Composite Module Analyst) algorithm for the purpose of searching for composite modules [28] in the promoters and enhancers of the Yes and No sets. We searched for a composite module consisting of a cluster of 10 TFs in a sliding window of 200–300 bp that statistically significantly separated sequences in the Yes and No sets (minimizing the Wilcoxon *p*-value).

Then, we searched for master regulator molecules in signal transduction pathways upstream of the identified TFs. The master regulator search used the TRANSPATH^®^ database (BIOBASE), release 2022.2 (geneXplain GmbH, Wolfenbüttel, Germany) (https://genexplain.com/transpath (accessed on December 2022)). A comprehensive signal transduction network of human cells was built by the software on the basis of reactions annotated in TRANSPATH^®^. All signal transduction reactions from TRANSPATH^®^ (including ligand binding reactions, phosphorylation and dephosphorylation reactions, complex formation reactions, ubiquitination, and other reactions known from the scientific literature) were considered as a weighted and directed graph. The main algorithm of the master regulator search has been described in earlier works [13,14]. The goal of the algorithm was to find nodes in the global signal transduction network that may potentially regulate the activity of a set of TFs found at the previous step of the analysis. Such nodes are considered as most promising drug targets, since any influence on such a node may switch the transcriptional programs of hundreds of genes that are regulated by their respective TFs. In our analysis, we ran the algorithm with a maximum radius of 12 steps upstream of each TF in the input set. The error rate of this algorithm is controlled by applying it 10,000 times to randomly generated sets of input transcription factors of the same set size. Then Z-score and FDR (false discovery rate) value of ranks were calculated for each potential master regulator node on the basis of such random runs (see detailed description in [22]). The error rate was controlled by an FDR threshold of 0.05.

## 5. Conclusions

The present *in vitro* study on methylation profiling identified epigenome-wide changes in the cells that represent the corresponding layers of the vein wall in response to oscillatory shear stress towards the endothelium. These epigenomic changes may be implicated in creating altered phenotypes of those cells, which reflects the morphological changes observed in incompetent veins (VVs). The master regulators that control the activity of key TFs regulating the genes near the differentially methylated sites were revealed for ECs, SMCs, and FBs. Due to the discovery of novel therapeutic targets, the future development of treatment strategies may eventually improve the quality of life of patients suffering from vascular diseases.

## Figures and Tables

**Table 1:** Top 20 genes among those significantly hypo- and hypermethylated in ECs upon exposure.

Gene Symbol	Gene Description	DiffScore ^1^	ECs + Avg_Beta ^2^	ECs − Avg_Beta	TargetID ^3^	*p*-Value ^4^
*EEF1D*	eukaryotic translation elongation factor 1 delta	−109.1854	0.6555644	0.8999788	cg22186533	1.21 × 10^−11^
*MMD*	monocyte to macrophage differentiation-associated	−35.18965	0.2981108	0.5100111	cg14861570	0.000302716
*TESK1*	testis associated actin remodeling kinase 1	−31.42026	0.4998818	0.6956196	cg09000510	0.000721064
*SLC24A4*	solute carrier family 24 member 4	−28.64084	0.3270859	0.5232344	cg15052901	0.001367464
*MYH3*	myosin heavy chain 3	−28.24431	0.2393593	0.4242639	cg18190433	0.001498197
*PDGFB*	platelet derived growth factor subunit B	−24.95273	0.11867	0.2644652	cg19167673	0.003196885
*AR*	androgen receptor	−24.15009	0.5732113	0.7435369	cg27271368	0.003845838
*ZCCHC10*	zinc finger CCHC-type containing 10	−22.23847	0.585928	0.748945	cg08801754	0.005972457
*CELSR3*	cadherin EGF LAG seven-pass G-type receptor 3	−22.23847	0.5043565	0.6789438	cg06621358	0.005972457
*CNIH2*	cornichon family AMPA receptor auxiliary protein 2	−21.25318	0.1661903	0.3161516	cg19026260	0.007493453
*ZFP28*	ZFP28 zinc finger protein	16.8081	0.4203998	0.2640518	cg23850212	0.02085403
*YY1*	YY1 transcription factor	18.57949	0.7956405	0.6552107	cg22763181	0.013869187
*FRMD1*	FERM domain containing 1	19.00112	0.6587915	0.49145	cg00350478	0.012586008
*MEST*	mesoderm specific transcript	21.27902	0.8093046	0.6647319	cg13917504	0.007449
*LARP1*	La ribonucleoprotein 1, translational regulator	23.04286	0.5051906	0.3232486	cg23613317	0.004962654
*CRELD2*	cysteine rich with EGF like domains 2	23.1515	0.836981	0.6953279	cg25882056	0.004840052
*RAB9A*	RAB9A, member RAS oncogene family	25.78106	0.7809207	0.6158033	cg02620228	0.002641764
*KRT17*	keratin 17	28.64084	0.7502007	0.5708218	cg00214794	0.001367464
*SLC29A1*	solute carrier family 29 member 1 (Augustine blood group)	45.2453	0.7506605	0.5342799	cg10519140	2.98862 × 10^−5^
*SLC45A1*	solute carrier family 45 member 1	57.2597	0.6744524	0.4233551	cg11283860	1.87945 × 10^−6^

^1^ DiffScore definition is given in Materials and Methods (4.2). ^2^ Avg_Beta represents an average methylation beta-value for ECs (+/− shear stress, respectively). ^3^ TargetID identifies the probe name. cg# represents CpG loci. ^4^ *p*-value = [10^(DiffScore/10)] for hypomethylated genes; [10^(−DiffScore/10)] for hypermethylated genes.

**Table 2:** Top 20 genes among those significantly hypo- and hypermethylated in SMCs upon exposure.

Gene Symbol	Gene Description	DiffScore ^1^	SMCs + Avg_Beta ^2^	SMCs − Avg_Beta	TargetID ^3^	*p*-Value ^4^
*SCT*	secretin	−44.15369	0.3001944	0.5255814	cg05782292	3.84265 × 10^−5^
*HLTF*	helicase-like transcription factor	−37.04874	0.3306479	0.5443499	cg26151310	0.0001973
*CDH2*	cadherin 2	−34.43425	0.3850524	0.5944616	cg09313439	0.000360226
*ZNRF1*	zinc and ring finger 1	−30.55943	0.385322	0.5856031	cg20957193	0.000879138
*PRADC1*	protease associated domain containing 1	−28.45422	0.4262078	0.6192294	cg26993951	0.001427506
*CCNJL*	cyclin J-like	−21.76985	0.4390145	0.6126426	cg17178888	0.006652961
*METTL25B*	methyltransferase-like 25B	−21.18877	0.4171189	0.590144	cg23881601	0.007605416
*CDCA5*	cell division cycle-associated 5	−21.18382	0.4315823	0.6038101	cg20252016	0.00761409
*SIRT3*	sirtuin 3	−20.95826	0.7362766	0.8588417	cg23530288	0.008019993
*TCF15*	transcription factor 15	−20.04535	0.3651721	0.534321	cg06143901	0.009896121
*TMEM215*	transmembrane protein 215	29.97724	0.8431917	0.6911527	cg11308840	0.001005254
*KMT2D*	lysine methyltransferase 2D	30.48615	0.4764972	0.2821061	cg24471867	0.000894098
*CDC37L1*	cell division cycle 37-like 1, HSP90 cochaperone	30.55943	0.8350812	0.6772436	cg22189519	0.000879138
*SPA17*	sperm autoantigenic protein 17	34.0979	0.8123621	0.6387361	cg22318304	0.000389233
*PGBD4*	piggyBac transposable element derived 4	35.79305	0.6302417	0.4193449	cg12237946	0.000263448
*KHK*	ketohexokinase	39.8873	0.8598925	0.6937668	cg01522194	0.000102629
*RNF213*	ring finger protein 213	44.15369	0.8767144	0.7105584	cg09907395	3.84265 × 10^−5^
*MRPL12*	mitochondrial ribosomal protein L12	48.63778	0.816642	0.6133671	cg01372689	1.36843 × 10^−5^
*RPRM*	reprimo, TP53-dependent G2 arrest mediator homolog	58.14957	0.6450388	0.3928624	cg18411898	1.53124 × 10^−6^E
*SLC22A18*	solute carrier family 22 member 18	77.42734	0.8139806	0.5674041	cg16035277	1.80828 × 10^−8^

^1^ DiffScore definition is given in Materials and Methods (4.2). ^2^ Avg_Beta represents an average methylation beta-value for SMCs (+/− shear stress, respectively). ^3^ TargetID identifies the probe name. cg# represents CpG loci. ^4^ *p*-value = [10^(DiffScore/10)] for hypomethylated genes; [10^(−DiffScore/10)] for hypermethylated genes.

**Table 3:** Top 20 genes among those significantly hypo- and hypermethylated in FBs upon exposure.

Gene Symbol	Gene Description	DiffScore ^1^	FBs + Avg_Beta ^2^	FBs − Avg_Beta	TargetID ^3^	*p*-Value ^4^
*PPP1R13L*	protein phosphatase 1 regulatory subunit 13-like	−287.4783	0.1425181	0.5611927	cg03554552	1.78719 × 10^−29^
*PPP1CC*	protein phosphatase 1 catalytic subunit gamma	−195.8575	0.5820595	0.9008529	cg03310453	2.59567 × 10^−20^
*SCRN2*	secernin 2	−174.5741	0.5376809	0.858446	cg11646887	3.48811 × 10^−18^
*MYL5*	myosin light chain 5	−174.3685	0.5000765	0.831169	cg18176712	3.65721 × 10^−18^
*FUCA1*	alpha-L-fucosidase 1	−166.3722	0.6312342	0.9132504	cg24792360	2.30558 × 10^−17^
*ISYNA1*	inositol-3-phosphate synthase 1	−161.8102	0.4622425	0.7933455	cg09374949	6.59144 × 10^−17^
*YIPF5*	Yip1 domain family member 5	−153.572	0.4413335	0.7704819	cg04293726	4.39339 × 10^−16^
*SEMA6C*	semaphorin 6C	−149.7812	0.4570227	0.7800868	cg25820693	1.05167 × 10^−15^
*FBN2*	fibrillin 2	−147.2405	0.7476242	0.9649287	cg25084878	1.88777 × 10^−15^
*RANGNRF*	RAN guanine nucleotide release factor	−146.2499	0.4167334	0.743665	cg11178443	2.37143 × 10^−15^
*DCAF16*	DDB1 and CUL4-associated factor 16	90.26163	0.7206882	0.4538092	cg27127056	9.41536 × 10^−10^
*SACM1L*	SAC1-like phosphatidylinositide phosphatase	91.75178	0.8458125	0.6134535	cg22986271	6.6807 × 10^−10^
*CACHD1*	cache domain containing 1	94.27448	0.7917484	0.5357191	cg19304410	3.73725 × 10^−10^
*UGDH*	UDP-glucose 6-dehydrogenase	97.15292	0.8846164	0.6643396	cg25117976	1.92623 × 10^−10^
*GGTLC2*	gamma-glutamyltransferase light chain 2	99.88158	0.8204779	0.5666317	cg01457622	1.02764 × 10^−10^
*C16orf54*	chromosome 16 open reading frame 54	101.7236	0.2649694	0.0511992	cg23093496	6.72419 × 10^−11^
*FXN*	frataxin	103.5087	0.5243153	0.2395225	cg23667933	4.4579 × 10^−11^
*RGS11*	regulator of G protein signaling 11	104.2319	0.4908251	0.2102013	cg01344518	3.77407 × 10^−11^
*PRICKLE3*	prickle planar cell polarity protein 3	105.7841	0.3453371	0.09902742	cg10417559	2.63992 × 10^−11^
*SPATA4*	spermatogenesis associated 4	335.9589	0.5617582	0.2024512	cg01311285	2.53577 × 10^−34^

^1^ DiffScore definition is given in Materials and Methods (4.2). ^2^ Avg_Beta represents an average methylation beta-value for FBs (+/− shear stress, respectively). ^3^ TargetID identifies the probe name. cg# represents CpG loci. ^4^ *p*-value = [10^(DiffScore/10)] for hypomethylated genes; [10^(−DiffScore/10)] for hypermethylated genes.

**Table 4:** Transcription factors of the predicted enhancer model potentially regulating the genes near the differentially methylated sites.

Gene Symbol	Gene Description	Regulatory Score ^1^	Yes-No Ratio ^2^
***ECs***			
*JUN*	Jun proto-oncogene, AP-1 transcription factor subunit	2.09	1.56
*CDX2*	caudal type homeobox 2	1.77	1.62
*POU2F1*	POU class 2 homeobox 1	1.61	1.87
*SREBF2*	sterol regulatory element binding transcription factor 2	1.59	1.33
*NKX3-1*	NK3 homeobox 1	1.58	1.58
*NHLH1*	nescient helix-loop-helix 1	1.39	2.6
*GABPA*	GA binding protein transcription factor subunit alpha	1.38	1.24
*RUNX3*	RUNX family transcription factor 3	1.32	4.61
*NRL*	neural retina leucine zipper	1.21	1.75
*MAFK*	MAF bZIP transcription factor K	1.15	2.13
***SMCs***			
*SREBF2*	sterol regulatory element binding transcription factor 2	1.8	1.5
*LEF1*	lymphoid enhancer binding factor 1	1.78	2.26
*IRF3*	interferon regulatory factor 3	1.76	2.39
*WT1*	WT1 transcription factor	1.74	5.13
*SFPQ*	splicing factor proline and glutamine rich	1.71	1.24
*POU2F1*	POU class 2 homeobox 1	1.7	3.54
*RBPJ*	recombination signal binding protein for immunoglobulin kappa J region	1.7	1.65
*DPF2*	double PHD fingers 2	1.64	1.31
*PAX5*	paired box 5	1.64	1.35
*SREBF1*	sterol regulatory element binding transcription factor 1	1.64	4.45
***FBs***			
*RELA*	RELA proto-oncogene, NF-kB subunit	2.27	1.9
*ESR1*	estrogen receptor 1	2.09	2.44
*TFAP2A*	transcription factor AP-2 alpha	1.66	2.12
*RUNX3*	RUNX family transcription factor 3	1.62	1.8
*ELF3*	E74 like ETS transcription factor 3	1.46	2.96
*TFAP2B*	transcription factor AP-2 beta	0	2.12
*TFAP2C*	transcription factor AP-2 gamma	0	2.42

^1^ Regulatory score is the measure of involvement of the given TF in controlling the expression of genes that encode the master regulators presented below (through positive feedback loops). ^2^ Yes–No ratio is the ratio between frequencies of the sites in Yes sequences versus No sequences. It describes the level of the enrichment of binding sites for the indicated TF in the regulatory target regions.

**Table 5:** Master regulators that may govern the regulation of genes near the differentially methylated sites.

Master Molecule Name	Gene Symbol	Gene Description	Total Rank ^1^
* **ECs** *			
PDGFB-isoform1(h)	*PDGFB*	platelet-derived growth factor subunit B	9
PDGFB-isoform2(h)	*PDGFB*	platelet-derived growth factor subunit B	9
PDGFB(h)	*PDGFB*	platelet-derived growth factor subunit B	11
HRS(h)	*HGS*	hepatocyte growth factor-regulated tyrosine kinase substrate	13
HRS:PtdIns(3)P:SARA:SMAD2	*HGS, SMAD2, ZFYVE9*	SMAD family member 2, hepatocyte growth factor-regulated tyrosine kinase substrate, zinc finger FYVE-type containing 9	13
HRS:PtdIns(3)P:SARA:SMAD3	*HGS, SMAD3, ZFYVE9*	SMAD family member 3, hepatocyte growth factor-regulated tyrosine kinase substrate, zinc finger FYVE-type containing 9	13
HRS-isoform1(h)	*HGS*	hepatocyte growth factor-regulated tyrosine kinase substrate	13
HRS(h){pY}	*HGS*	hepatocyte growth factor-regulated tyrosine kinase substrate	13
HRS-isoform2(h)	*HGS*	hepatocyte growth factor-regulated tyrosine kinase substrate	13
HRS(h){pY334}	*HGS*	hepatocyte growth factor-regulated tyrosine kinase substrate	13
* **SMCs** *			
HRS(h)	*HGS*	hepatocyte growth factor-regulated tyrosine kinase substrate	30
HRS:PtdIns(3)P:SARA:SMAD2	*HGS, SMAD2, ZFYVE9*	SMAD family member 2, hepatocyte growth factor-regulated tyrosine kinase substrate, zinc finger FYVE-type containing 9	30
HRS:PtdIns(3)P:SARA:SMAD3	*HGS, SMAD3, ZFYVE9*	SMAD family member 3, hepatocyte growth factor-regulated tyrosine kinase substrate, zinc finger FYVE-type containing 9	30
HRS-isoform1(h)	*HGS*	hepatocyte growth factor-regulated tyrosine kinase substrate	30
HRS(h){pY}	*HGS*	hepatocyte growth factor-regulated tyrosine kinase substrate	30
HRS-isoform2(h)	*HGS*	hepatocyte growth factor-regulated tyrosine kinase substrate	30
HRS(h){pY334}	*HGS*	hepatocyte growth factor-regulated tyrosine kinase substrate	30
Sprouty2(h){p}	*SPRY2*	sprouty RTK signaling antagonist 2	30
Sprouty2(h)	*SPRY2*	sprouty RTK signaling antagonist 2	31
CD23(h)	*FCER2*	Fc fragment of IgE receptor II	32
* **FBs** *			
SOCS-1(h)	*SOCS1*	suppressor of cytokine signaling 1	21
p50:NF-kappaBp65:SOCS-1	*NFKB1, RELA, SOCS1*	RELA proto-oncogene, NF-kB subunit, nuclear factor kappa B subunit 1, suppressor of cytokine signaling 1	21
p50:NF-kappaBp65{ub}n:SOCS-1	*NFKB1, RELA, SOCS1*	RELA proto-oncogene, NF-kB subunit, nuclear factor kappa B subunit 1, suppressor of cytokine signaling 1	21
SOCS-1(h)	*SOCS1*	suppressor of cytokine signaling 1	29
IGF-2R(h)	*IGF2R*	insulin like growth factor 2 receptor	32
G12/13	*GNA12, GNA13, GNB1, GNB1L, GNB2, GNB3, GNB4, GNB5, GNG2, GNG3, GNG5, GNGT1*	G protein subunit alpha 12, G protein subunit alpha 13, G protein subunit beta 1, G protein subunit beta 1 like, G protein subunit beta 2, G protein subunit beta 3, G protein subunit beta 4, G protein subunit beta 5, G protein subunit gamma 2, G protein subunit gamma 3, G protein subunit gamma 5, G protein subunit gamma transducin 1	49
DHTXisoform1(h)	*UVRAG*	UV radiation resistance associated	50
F8B(h){gly}n	*F8*	coagulation factor VIII	51
F8B(h)	*F8*	coagulation factor VIII	52
F8B-isoform2(h)	*F8*	coagulation factor VIII	53

^1^ Total rank is the sum of the ranks of the master molecules sorted by keynode score, CMA score, and epigenomics data. The lower the total rank is, the more likely it is that a master molecule is considered as a master regulator.

## Data Availability

Data are contained within the article and Supplementary Materials.

## Supplementary Materials

The following supporting information can be downloaded at: https://www.mdpi.com/article/10.3390/epigenomes7010008/s1, Figure S1: Cluster heatmap depicting methylation levels of the top 20 genes among those significantly hypo- and hypermethylated in 3 cell types (combined) upon the exposure; Figure S2: Enriched TRANSPATH^®^ Pathways (2022.2) of genes near differentially methylated sites in ECs; Figure S3: Enriched TRANSPATH^®^ Pathways (2022.2) of genes near differentially methylated sites in SMCs; Figure S4: Enriched TRANSPATH^®^ Pathways (2022.2) of genes near differentially methylated sites in FBs; Figure S5: Cell culture examples; Figure S6: Experiment design; Table S1: DiffMethylated genes_FDR_DiffScore 13_ECs; Table S2: DiffMethylated genes_FDR_DiffScore 13_SMCs; Table S3: DiffMethylated genes_FDR_DiffScore 13_FBs; Table S4: GO (biological process)_ECs; Table S5: GO (biological process)_SMCs; Table S6: GO (biological process)_FBs; Table S7: TRANSPATH Pathways (2022.2)_ECs; Table S8: TRANSPATH Pathways (2022.2)_SMCs; Table S9: TRANSPATH Pathways (2022.2)_FBs; Table S10: Transcription factor proteins annotated_ECs; Table S11: Transcription factor proteins annotated_SMCs; Table S12: Transcription factor proteins annotated_FBs; Table S13: Keynodes for best model annotated ranked_Master regulators_ECs; Table S14: Keynodes for best model annotated ranked_Master regulators_SMCs; Table S15: Keynodes for best model annotated ranked_Master regulators_FBs; Table S16: Feedback Controlled Master Regulators_ECs; Table S17: Feedback Controlled Master Regulators_SMCs; Table S18: Feedback Controlled Master Regulators_FBs.
