# Supplementary material for: Epigenome-Wide Changes in the Cell Layers of the Vein Wall When Exposing the Venous Endothelium to Oscillatory Shear Stress
Source: Epigenomes. 2023 Mar 20;7(1):8. doi: 10.3390/epigenomes7010008 (PMC10048778; doi:10.3390/epigenomes7010008)
Supplement: Supplementary file 1 [file epigenomes-07-00008-s001.zip › Supplementary Figures.pdf]

Supplementary Figures

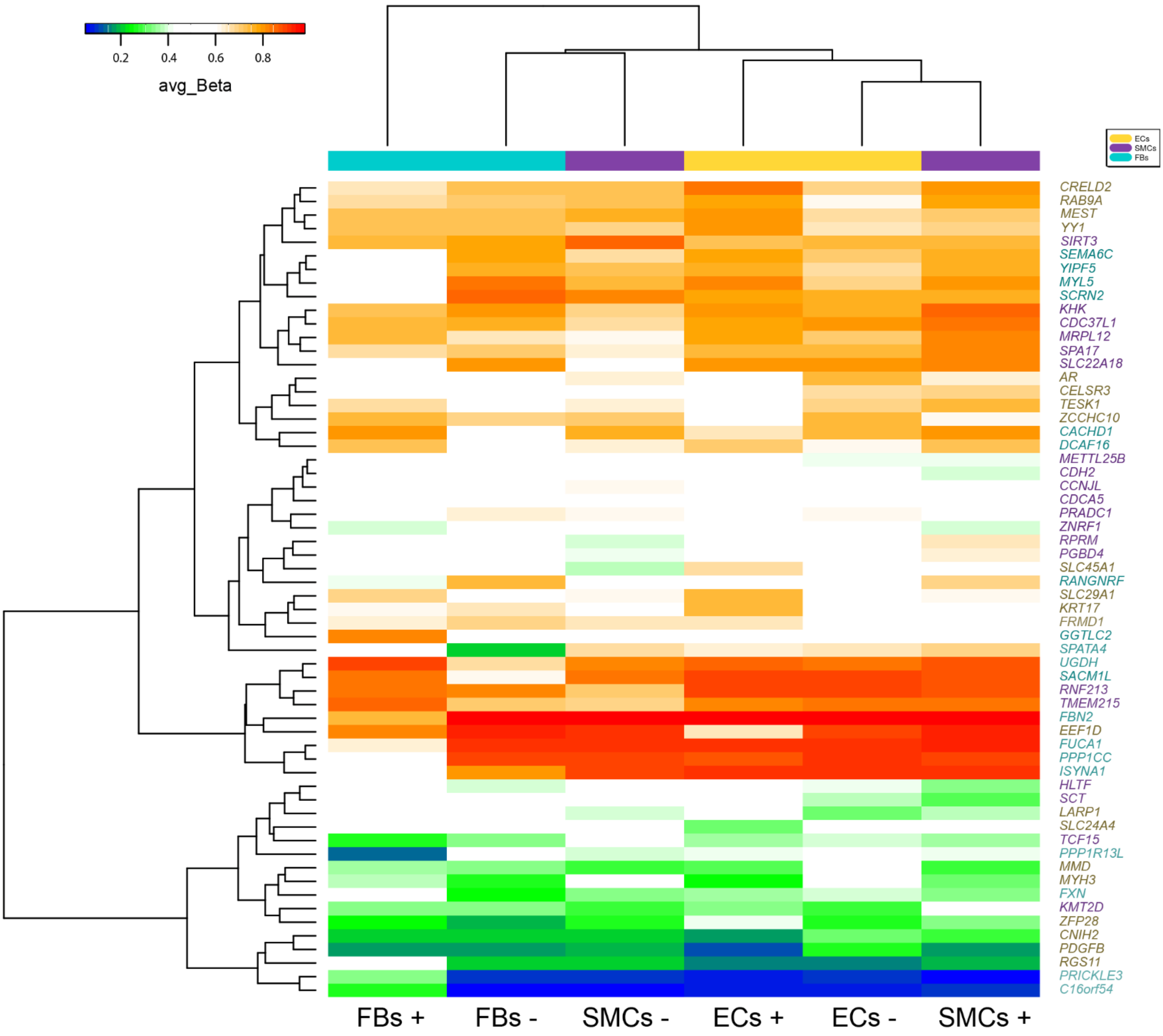

Figure S1. Cluster heatmap depicting methylation levels of the top 20 genes among those significantly hypo- and hypermethylated in 3 cell types (combined) upon exposure. Heatmap displays differentially methylated genes: ranging from hypomethylated (blue) to hypermethylated (red). avg_Beta represents an average methylation beta value and corresponds to a certain color within the range. Cluster patterns are shown on the left and the upper sides of the diagram. On the right side of the diagram, the names of genes differentially methylated in ECs are written in beige, in SMCs - in purple, in FBs - in turquoise.

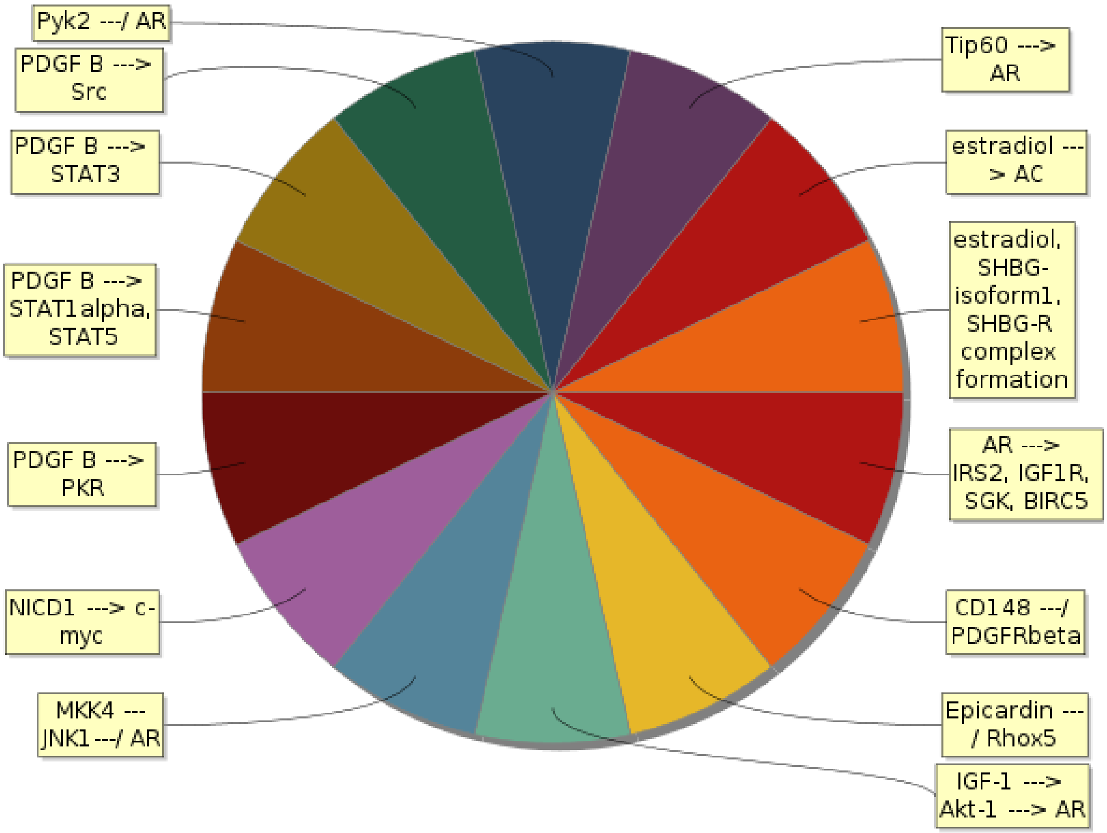

Figure S2. Enriched TRANSPATH® Pathways (2022.2) of genes near differentially methylated sites in ECs.

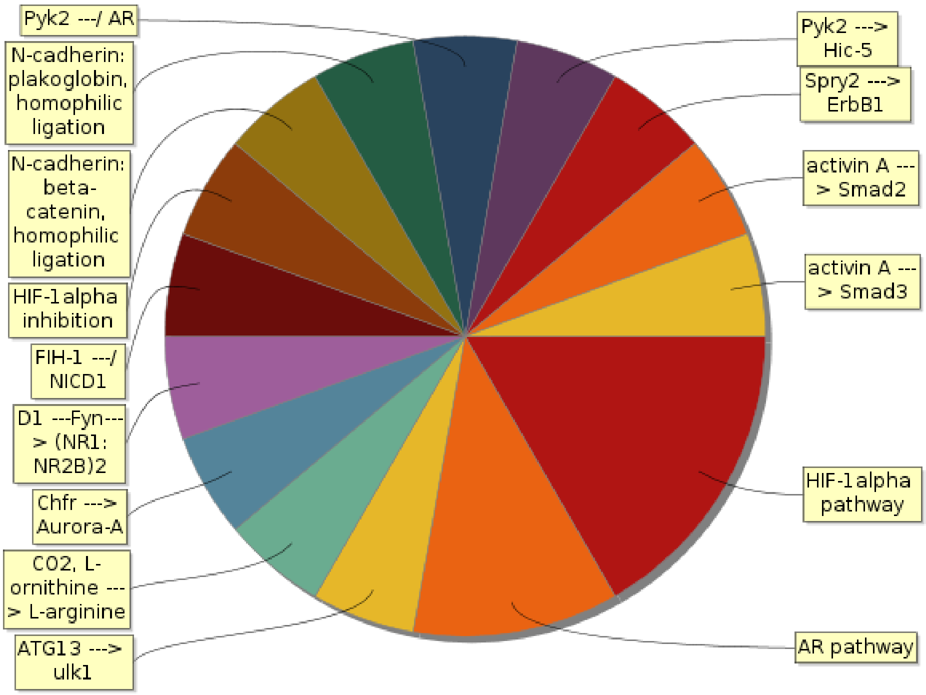

Figure S3. Enriched TRANSPATH® Pathways (2022.2) of genes near differentially methylated sites in SMCs.

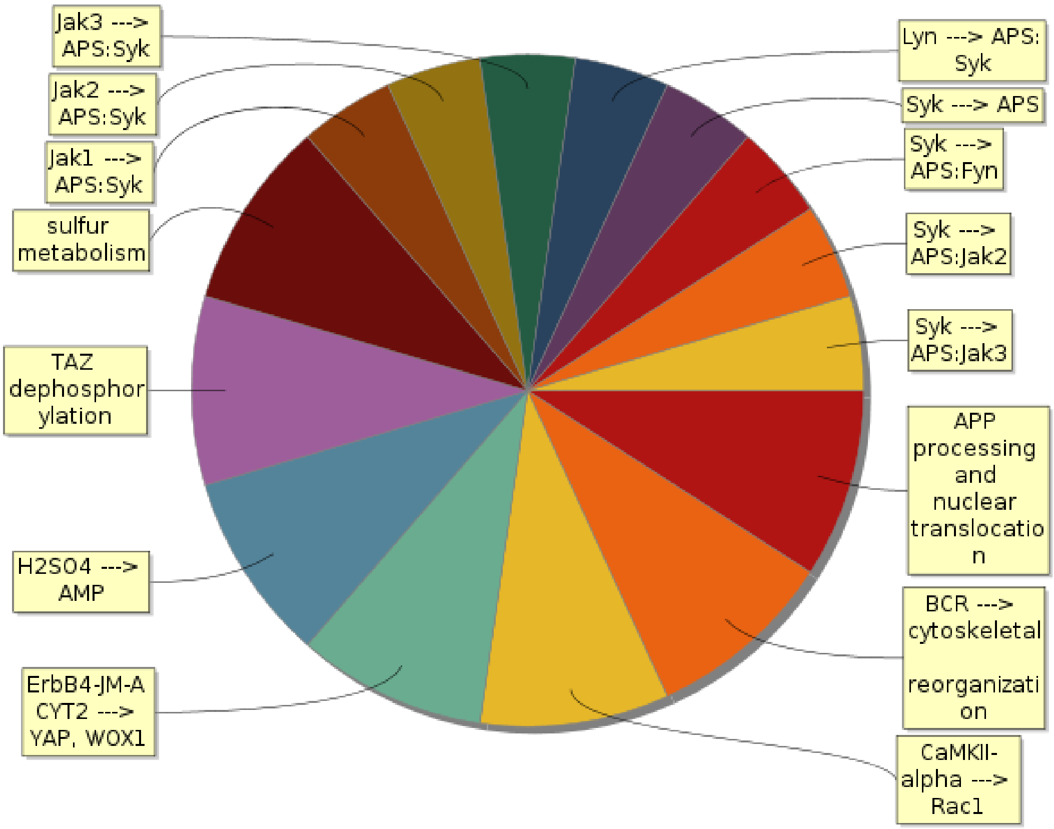

Figure S4. Enriched TRANSPATH® Pathways (2022.2) of genes near differentially methylated sites in FBs.

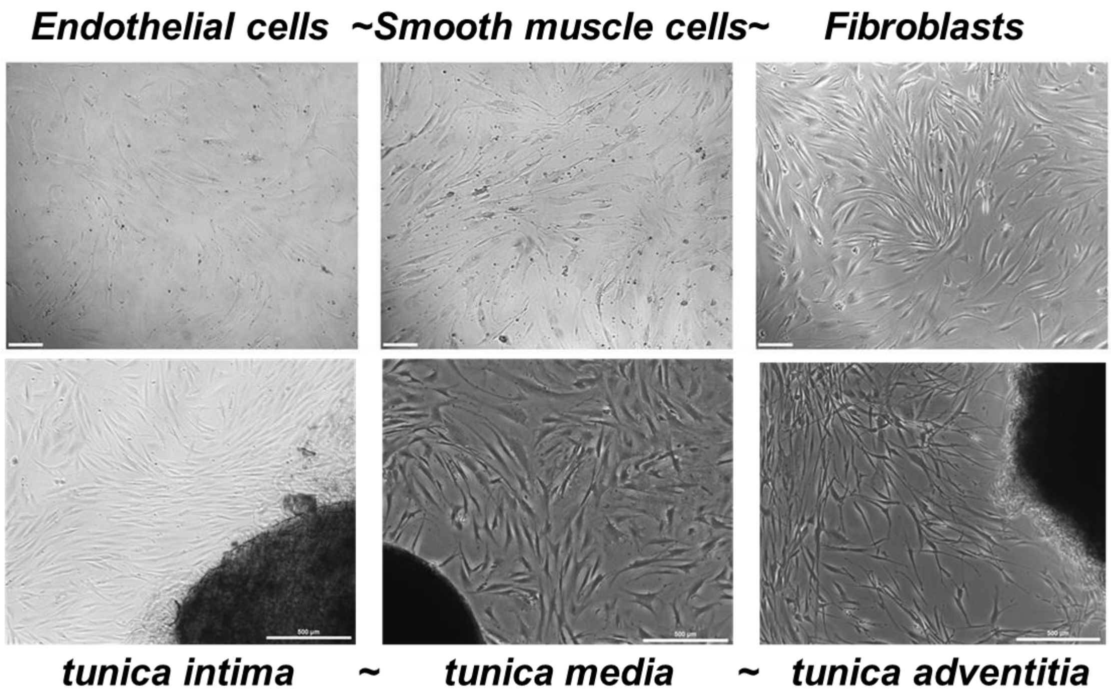

Figure S5. Cell culture examples. Morphology of cells (representing 3 layers of the venous wall) grown in selective media from non-varicose vein segments (dark spots). Phase contrast, upper row: 100µm ruler, lower row: 500µm ruler.

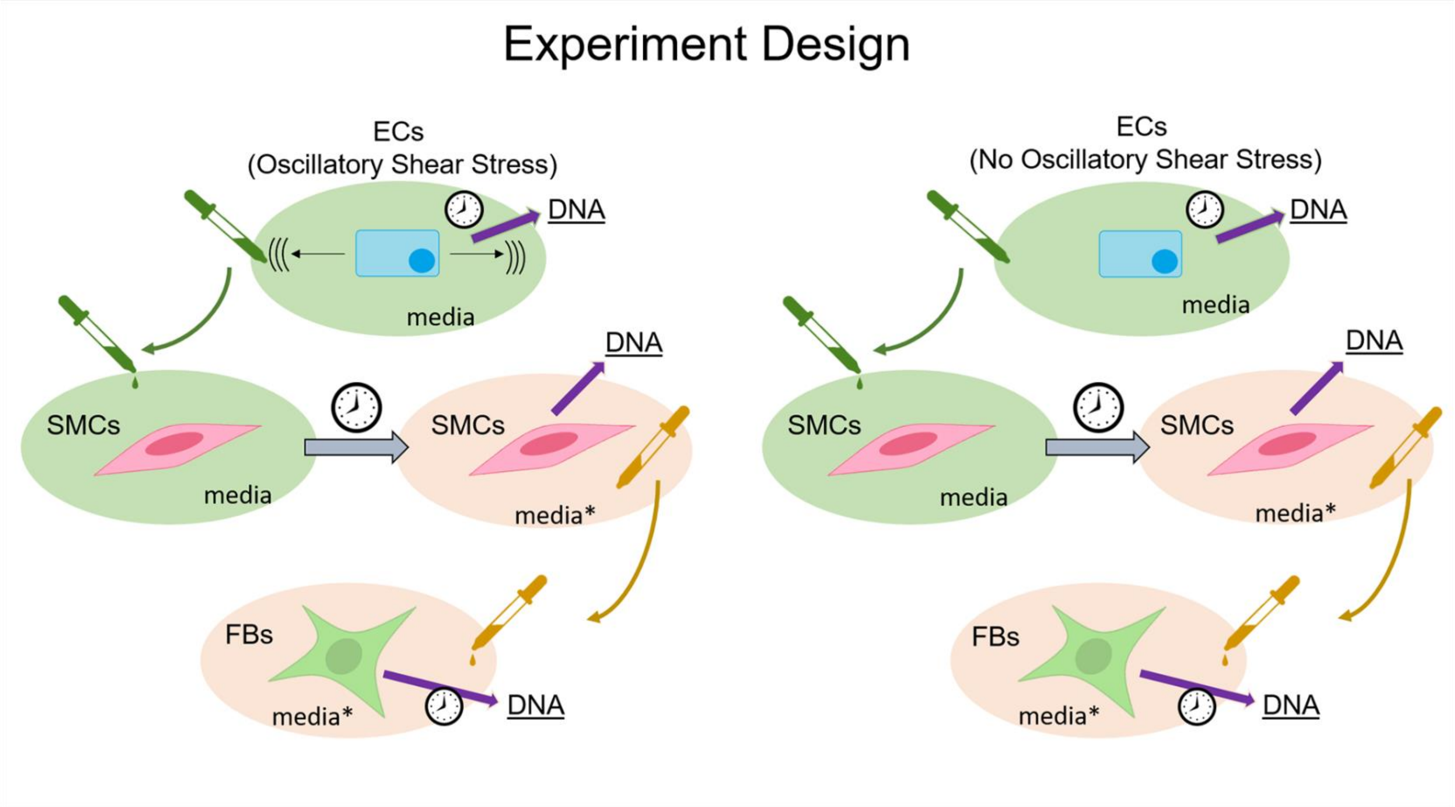

Figure S6. Experiment design. Each experimental condition was performed in triplicate.
